# Prognostic Indicators of Severe Dengue Infection in Adult Patients in Thailand

**DOI:** 10.3390/tropicalmed10080233

**Published:** 2025-08-18

**Authors:** Patcharin Khamnuan, Surangrat Pongpan, Pantitcha Thanatrakolsri, Supa Vittaporn, Punnaphat Daraswang, Sirawan Samsee

**Affiliations:** 1 Faculty of Public Health, Thammasat University, Lampang 52190, Thailand; patcharin.k@fph.tu.ac.th (P.K.); pantitcha.o@fph.tu.ac.th (P.T.); supa.v@fph.tu.ac.th (S.V.); 2 Thammasat University Research Unit in Environment, Health and Epidemiology, Lampang 52190, Thailand; 3 Buriram Hospital, Muang, Buriram 31000, Thailand; punnapath.da@cpird.in.th; 4 Sisaket Hospital, Muang, Sisaket 33000, Thailand; sirawansam.2560@gmail.com

**Keywords:** prognostic factors, severe dengue, dengue infection, dengue shock syndrome, clinical risk, vector borne diseases

## Abstract

Background: Dengue infection is a spreading vector borne disease with most severe infection-related fatalities occurring in adults. This study was conducted to explore prognostic indicators of dengue infection severity. Methods: This study included patients aged over 15 years who were diagnosed with dengue viral infection. Data were collected from nine hospitals across all regions of Thailand between January 2019 and December 2022. Diagnosis of dengue infection was confirmed by a positive result for the NS-1 antigen via RT–PCR, IgM antibody, or IgG antibody tests. Data including gender, age, BMI, underlying disease, clinical characteristics and laboratory findings were collected. Multivariable logistic regression with backward elimination was used to identify a set of prognostic factors. Results: The prognostic indicators of severe dengue were age < 55 years (OR = 6.13, *p* = 0.054), severe bleeding (bleeding from the gastrointestinal tract, hematemesis, melena, menorrhagia, or hematuria) (OR = 20.75, *p* < 0.001), pleural effusion (OR = 10.23, *p* < 0.001), and platelet ≤ 100,000 (/µL) (OR = 3.62, *p* = 0.035). These predictors were able to accurately estimate the severity of dengue infection with an area under the receiver operating curve (AuROC) of 0.836. Conclusions: The proposed four prognostic factors can be applied to predict severe dengue infections. These findings may inform the development of a risk scoring system to forecast severe dengue infection, early detection, and appropriate treatment during sickness.

## 1. Introduction

Dengue fever is a pressing public health concern in Thailand, with recent outbreaks reaching historically high levels. According to the World Health Organization, the country experienced a dramatic resurgence in 2023—an over 300% increase from 46,678 reported cases in 2022 to approximately 136,655 cases, with more than 122 deaths by October [1].

This trend continued into early 2024, with 16,319 cases and 16 deaths reported as of February, nearly doubling the numbers from the same period in 2023 [2]. This resurgence has been partly driven by climate anomalies, such as El Niño events and prolonged rainy seasons, which create ideal breeding conditions for Aedes mosquitoes—the primary vector for dengue transmission [3].

At the molecular level, secondary infection with a different dengue virus serotype can lead to antibody-dependent enhancement (ADE), promoting viral replication and contributing to severe disease progression [4,5]. Severe dengue cases are also associated with dysregulated immune responses, including cytokine storms, monocyte dysfunction, and impaired interferon signaling. Additionally, viral proteins such as NS5 facilitate immune evasion and induce metabolic reprogramming of host cells [4,5,6]. Together, these mechanisms highlight the complex virus–host interactions that underlie clinical severity and outcomes in dengue infection.

Most dengue virus (DENV) infections are asymptomatic. When symptoms do occur, they commonly include high-grade fever, retro-orbital pain, myalgia, arthralgia, rash, nausea, and vomiting. In most patients, symptoms resolve within one to two weeks. However, a small proportion progress to severe dengue, characterized by plasma leakage, bleeding, and organ dysfunction, which can be life-threatening without timely intervention [7,8].

Secondary infection with a heterologous DENV serotype has been shown to increase the risk of progressing to severe dengue due to immunological mechanisms such as antibody-dependent enhancement (ADE) [9,10]. The main causes of death from dengue infection include prolonged shock, massive bleeding, and fluid overload. Dengue can be fatal in severe cases if it is not identified and treated immediately [11].

Previous studies have identified numerous clinical and laboratory parameters that are associated with severe dengue infection (Table 1).

We identified variables using basic clinical laboratory data in routine practice obtained on the day of admission, including demographic, clinical presentation, hemodynamic, hematological, and biochemical laboratory parameters. This study aimed to identify clinical and laboratory markers that can predict severe dengue fever in adult patients upon arrival at hospital emergency departments or outpatient clinics.

## 2. Materials and Methods

### 2.1. Study Design and Study Areas

Nine hospitals in Thailand, including Tak Hospital, Nakhon Pathom Hospital, Phra Nakhon Si Ayutthaya Hospital, Rayong Hospital, Sisaket Hospital, Surin Hospital, Loei Hospital, Trang Hospital, and Phatthalung hospital participated in conducting a case–control study.

### 2.2. Study Population

A confirmed case of dengue virus infection in a patient over 15 years of age was defined as a positive result from one or more of the following diagnostic methods: NS–1 antigen, RT–PCR, IgM antibody test, or IgG antibody test. A positive IgM result was considered indicative of an acute or recent infection, while a positive IgG result alone, without accompanying IgM, was not sufficient for case confirmation. Diagnostic tests could be used individually or in combination, depending on availability at the participating hospitals. All patients were classified into 2 groups using the following criteria modified from WHO criteria in 2009 [27]: mild and severe dengue infection.

Dengue cases were identified between 2019 and 2022 using diagnostic codes based on the International Statistical Classification of Diseases and Related Health Problems, 10th Revision (ICD-10), including A90 (Dengue fever), A91 (Dengue hemorrhagic fever), A97.0 (Dengue without warning signs), A97.1 (Dengue with warning signs), and A97.2 (Severe dengue) [28].

In this study, the term “adult” refers to patients aged 15 years and older, based on clinical practice guidelines commonly used in Thailand.

### 2.3. Definition of Dengue Severity

Severe group (Case): The case definition was considered fulfilled if at least one of the following two criteria was present:

(1) Severe fluid buildup and/or plasma leakage that can cause shock or respiratory dis-tress. Any one of the following symptoms (i.e., only one criterion is required) could indicate shock: signs of respiratory distress, narrow pulse pressure, or hypotension relative to age.
(2) Severe organ impairment including liver failure, renal failure, encephalopathy, dis-seminated intravascular coagulation, and pulmonary edema.

Non-severe group (Control):

Patients who did not meet any of the criteria of a severe dengue infection.

### 2.4. Study Size Estimation

We estimated the sample size to achieve 80% power at a 5% significance level (two-sided) with a 4:1 ratio of non-severe to severe dengue cases. The required sample size was calculated based on clinical experience and data availability.

### 2.5. Indicator Parameters

Data collection included the following parameters:

(1) Baseline characteristics data: gender, age, BMI, underlying disease.
(2) Clinical characteristics: fever, headache, myalgia, retro-orbital pain, bone pain, joint pain, abdominal pain, vomiting, cough, diarrhea, petechiae, rash, epistaxis, bleeding from the gums, severe bleeding, hepatomegaly, pleural effusion, swift, feeble pulse, systolic blood pressure (SBP), diastolic blood pressure (DBP), pulse pressure.
(3) Laboratory findings: hemoglobin, hematocrit (HCT), platelets, white blood cells (WBC), neutrophils, lymphocytes, alanine aminotransferase (ALT), aspartate aminotransferase (AST).

All clinical and laboratory variables were collected on the day of hospital admission, prior to the final classification of severe dengue.

### 2.6. Data Analysis

In our first model, we identified predictors associated with critical severe dengue. Next, we examined the univariate associations between each independent variable and dengue severity using appropriate statistical tests. For the final model, multivariable logistic regression was conducted using backward elimination to identify independent predictors of severe dengue. The results are presented as odds ratios (ORs) with 95% confidence intervals (CIs). An area under the receiver operating characteristic curve (AuROC) was used to determine the predictive ability, with a cutoff value selected in the range of 0.7 to 0.8, representing an acceptable to good level of predictivity according to commonly accepted diagnostic standards.

**Figure 1:**
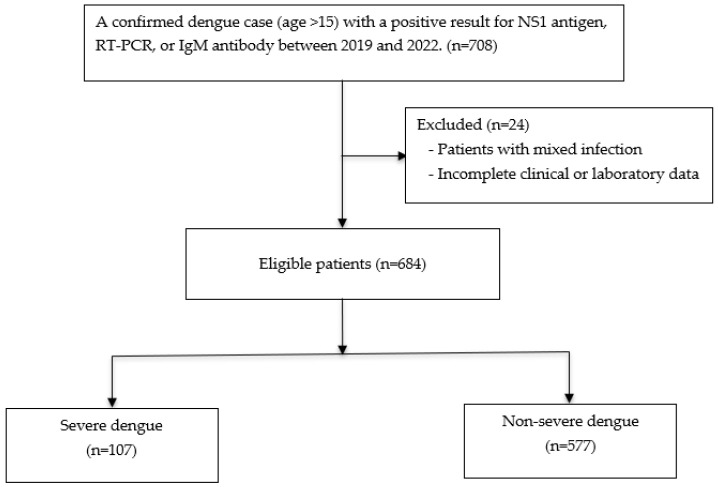
Study flow diagram of patient selection and stratification into severe and non-severe dengue groups.

The diagram illustrates the selection process of patients with confirmed dengue infection (NS1 antigen, RT-PCR, IgM, or IgG positive) from nine hospitals in Thailand between January 2019 and December 2022. Patients with mixed infection and those with incomplete clinical or laboratory data were excluded. Eligible patients were classified into two groups according to the modified WHO 2009 criteria: severe dengue (n = 107) and non-severe dengue (n = 577). (Figure 1)

### 2.7. Ethical Considerations

Board of Institutional Review Statement: The Human Research Ethics Committee of Thammasat University (Science) and nine hospitals approved this study in accordance with the Belmont Report, the International Practice (ICH-GCP), the Declaration of Helsinki, and CIOMS guidelines.

## 3. Results

### 3.1. Patient Characteristics

Patients were categorized into two groups, severe (n = 107) and non-severe (n = 577), following the criteria. There were statistically significant differences in headache, abdominal pain, bleeding episodes, pleural effusion, rapid weak pulse, systolic and diastolic blood pressure, pulse pressure, platelets, AST, and ALT between the severe and non-severe dengue groups (Table 2).

### 3.2. Factors Associated with Severe Dengue Hemorrhagic Infection

In the univariable logistic regression analysis, several clinical and laboratory variables were significantly associated with severe dengue, including severe bleeding, pleural effusion, platelet ≤ 100,000/µL, AST > 40 U/L, ALT > 40 U/L, and low blood pressure. In the multivariable analysis, only a limited number of variables remained independent prognostic indicators: severe bleeding (adjusted OR = 20.75, 95% CI 10.66–40.38, *p* < 0.001), pleural effusion (adjusted OR = 10.23, 95% CI 4.65–22.54, *p* < 0.001), and platelet ≤ 100,000/µL (adjusted OR = 3.62, 95% CI 1.10–11.94, *p* = 0.035). This indicates that while many variables showed significant crude associations with severe dengue, only these three factors were independently associated with disease severity after adjusting for other covariates. Notably, age < 55 years demonstrated a borderline association in the multivariable model (adjusted OR = 6.13, 95% CI 0.97–38.87, *p* = 0.054), suggesting a potential trend toward significance (Table 3).

**Figure 2:**
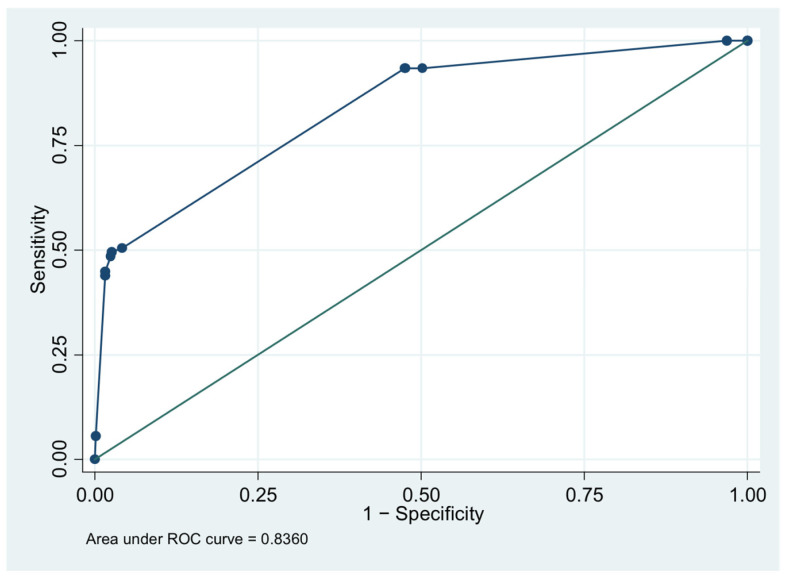
Receiver operating characteristic (ROC) curve of the multivariable logistic regression.

The model for predicting severe dengue infection includes four combined predictors: age < 55 years, severe bleeding, pleural effusion, and platelet ≤ 100,000/µL. The y-axis represents sensitivity, and the x-axis represents 1 − specificity. The area under the ROC curve (AuROC) was 0.8360 (Figure 2).

The blue line represents the ROC curve of the multivariable logistic regression model, showing the relationship between sensitivity and 1 – specificity, and the green line represents the reference line (diagonal line) indicating no discriminative ability.

## 4. Discussion

Age < 55 years was associated with higher odds of severe dengue in our analysis and this result showed borderline significance (*p* = 0.054) and contrasts with many prior studies identifying older age as a risk factor [14]. This unexpected finding may reflect specific demographic characteristics of the study population and should be interpreted with caution.

Moreover, variations in the population’s serological immunity in dengue-endemic areas may help explain this unexpected association. Another explanation could be variations in dengue genotypes; however, structural differences between dengue viruses have been demonstrated to correlate with pathogenesis, and a number of studies have already indicated that variations in dengue virus strains may be important in determining the severity of the disease. Adults and older children in endemic areas are more likely to have previously contracted dengue, and there is also a higher chance of developing a secondary infection and consequently a serious infection [20].

Although population-level differences in dengue immunity could contribute to the variability in disease severity, we did not obtain individual data on prior dengue infections.

The apparent association between younger age and severity in this study (*p* = 0.054) should be interpreted with caution, as this finding does not demonstrate a robust causal relationship and requires further confirmation in future studies that include serological or clinical history of previous dengue episodes.

One of the side effects of dengue fever that arises from plasma leakage into the pleural cavity is pleural effusion [29]. Pleural effusion is a recognized clinical complication resulting from plasma leakage in severe dengue infection, and has been consistently reported as a strong predictor of disease severity. Pleural effusion and pulmonary edema are also consistent with DF in the pathophysiology of dengue. About 38.6% of severe dengue cases present with pleural effusion caused by plasma leakage [30]. Previous studies’ findings are consistent with our study in supporting the prediction that pleural effusion has a close relationship with severe dengue [13,14,16,18,31].

In dengue patients, spontaneous bleeding is a relatively common complication that is associated with increased mortality [32]. Severe bleeding is strongly associated with SD, including hematemesis, melena, gum bleeding, and epistaxis [32]. These findings are consistent with those of Gulati S et al., who demonstrated that DHF frequently manifests as hemorrhages and the potentially fatal shock syndrome [33].

Evidence suggests that the dengue virus induces pathophysiological alterations in all hemostasis components, leading to abnormal von Willebrand factor (VWF) multimers, vasculopathy, thrombocytopenia, thrombopathy, reduction of several coagulation factors, increased antifibrinolytic factors, and consumption of natural anticoagulants. Only in extreme dengue cases can profound disseminated intravascular coagulation occur; this complication results in uncontrollable bleeding and death [34].

From primary to tertiary healthcare settings, hematological analyses have been used for dengue diagnosis and severity classification. In our study, the platelet count was substantially correlated with the course of the disease. Individuals with low platelet counts are more likely to experience severe disease.

We acknowledge that bleeding and pleural effusion are part of the SD classification criteria, which may introduce a tautological bias when used as prognostic indicators. Although these variables were recorded at admission prior to classification, their role as part of the SD diagnostic criteria may introduce tautological bias, which should be considered when interpreting the predictive model. Future work should account for this bias methodologically.

Additionally, although platelet count was found to be significantly associated with SD in our model, we did not assess its direct relationship with bleeding. This limitation, as well as the lack of serial platelet measurements before SD onset, is noted and should be considered in future studies.

## 5. Conclusions

The findings revealed that age under 55 years, severe bleeding, pleural effusion, and thrombocytopenia (platelet ≤ 100,000/µL) were significantly associated with the progression to severe dengue. These predictors demonstrated good accuracy, with an AuROC of 0.836, and may serve as practical clinical indicators for early risk stratification. Incorporating these factors into initial assessment protocols in emergency or outpatient settings may facilitate timely interventions, ultimately reducing the risk of complications, morbidity, and mortality in dengue patients.

## Figures and Tables

**Table 1:** Summary of predictors associated with severe dengue infection.

Category	Predictive Factor	References
Demographic	Female gender	[12,13]
	Age over 40–65 years	[14]
	Obesity	[15]
Clinical Symptoms	Hepatomegaly	[16]
	Abdominal pain and/or tenderness	[13,16,17]
	Vomiting	[16,18]
	Restlessness	[16]
	Dyspnea	[16]
	Impaired consciousness	[16]
	Low systolic blood pressure	[18]
	Pulse pressure < 20 mmHg	[12]
	Severe bleeding (e.g., hematemesis, melena, epistaxis, bleeding from the gums)	[13,16,17,19,20]
Comorbidities	Diabetes mellitus	[13,14]
	Hypertension	[13,15]
	Hyperlipidemia	[15]
	Kidney disease	[13]
	Cardiovascular disease	[13,14]
	Asthma	[14]
	≥2 comorbidities	[14,15]
	Secondary infection	[13,16]
Signs of Plasma Leakage	Ascites	[16]
	Pleural effusion	[13,14,16,18]
Laboratory Findings	Leukopenia	[21]
	Thrombocytopenia	[13,14,15,16,19,21,22]
	Increased hematocrit	[14,16,19]
	Elevated AST and/or ALT	[12,13,15,16,17,22,23,24,25]
	Low albumin	[13,26]
Radiologic Finding	Gallbladder wall thickening	[16]

**Table 2:** Demographic and clinical manifestations of patients with severe and non-severe dengue infection.

Patient Characteristics	Severe	Non-Severe	*p*-Value
	n (%)	n (%)	
Demographic			
Female	54 (50.5)	260 (45.06)	0.342
Age (years), mean (SD)	27.1 (±10.8)	28.9 (±13.1)	0.125
BMI (kg/m^2^)	22.4 (4.9)	23.3 (5.1)	0.079
Underlying disease	5 (4.7)	45 (7.8)	0.315
Hypertension	2 (1.9)	28 (4.9)	0.206
Diabetes mellitus	3 (2.8)	19 (3.3)	1.000
Dyslipidemia	1 (0.9)	16 (2.8)	0.495
Asthma	1 (0.9)	7 (1.2)	1.000
Clinical presentation			
Fever	107 (100)	577 (100)	-
Headache	60 (56.1)	384 (66.6)	0.047
Myalgia	70 (77.0)	121 (74.7)	0.163
Retro-orbital pain	15 (14.0)	77 (13.3)	0.877
Bone pain	4 (3.7)	8 (1.4)	0.102
Joint pain	5 (4.7)	40 (6.9)	0.524
Abdominal pain	34 (31.8)	110 (19.1)	0.139
Vomiting	56 (52.3)	255 (44.2)	0.323
Cough	21 (19.6)	141 (24.4)	0.139
Diarrhea	22 (20.6)	119 (20.6)	1.000
Petechiae	19 (17.8)	87 (15.1)	0.469
Rash	4 (3.7)	44 (7.6)	0.214
Epistaxis	5 (4.7)	25 (4.3)	0.800
Bleeding from the gums	10 (9.4)	54 (9.4)	1.000
Severe bleeding *	50 (46.7)	19 (3.3)	<0.001
Hepatomegaly	3 (2.8)	6 (1.0)	0.154
Pleural effusion	11 (10.3)	7 (1.2)	<0.001
Rapid, weak pulse	40 (37.4)	0	<0.001
Hemodynamics, mean (SD)			
Systolic blood pressure (mmHg)	89.6 (17.8)	114.7 (11.9)	<0.001
Diastolic blood pressure (mmHg)	56.4 (14.4)	72.0 (10.0)	<0.001
Pulse pressure	33.2 (9.6)	42.8 (10.3)	<0.001
Hematological			
Hematocrit (%), mean (SD)	42.2 (5.3)	42.2 (7.6)	0.880
White blood cell (/µL), median (IQR)	3790 (2600, 5600)	3500 (2600, 5000)	0.569
Platelet (/µL), median (IQR)	48 (20, 73)	103 (71, 151)	<0.001
Neutrophils (%), mean (SD)	59.4 (19.7)	61.2 (16.9)	0.334
Lymphocyte (%), median (IQR)	25.7 (16, 36.3)	25 (17, 37)	0.817
Biochemical			
AST (U/L), median (IQR)	122 (67.5, 385)	83 (44, 133)	<0.001
ALT (U/L), median (IQR)	68 (34.5, 211.5)	45 (26, 89)	<0.001
Duration of admission (days), median (IQR)	4 (3, 5)	3 (2, 4)	0.001
In hospital death	6 (5.6)	0	<0.001

* Bleeding from the gastrointestinal tract, hematemesis, melena, menorrhagia, hematuria. Abbreviations: IQR, interquartile range; AST, aspartate aminotransferase; ALT, alanine aminotransferase.

**Table 3:** Univariable and multivariable logistic regression analysis of prognostic indicators for severe dengue in adult patients.

Prognostic Indicators	Crude OR (95% CI)	*p*-Value	Adjusted OR (95% CI)	*p*-Value
Age < 55 years	3.29 (0.78–13.89)	0.106	6.13 (0.97–8.87)	0.054
Female	1.24 (0.82–1.88)	0.342	–	–
BMI ≥ 25 kg/m^2^	0.80 (0.49–1.31)	0.381	–	–
Underlying disease	0.58 (0.22–1.50)	0.259	–	–
Hypertension	0.37 (0.09–1.59)	0.183	–	–
Diabetes mellitus	0.85 (0.25–2.91)	0.792	–	–
Dyslipidemia	0.33 (0.04–2.52)	0.286	–	–
Asthma	0.77 (0.09–6.31)	0.806	–	–
Fever ≥ 38.0 °C	0.21 (0.13–0.33)	<0.001	–	–
Headache	0.64 (0.42–0.98)	0.047	–	–
Myalgia	0.73 (0.47–1.12)	0.152	–	–
Retro-orbital pain	1.06 (0.58–1.92)	0.851	–	–
Bone pain	2.76 (0.82–9.34)	0.102	–	–
Joint pain	0.66 (0.25–1.71)	0.390	–	–
Abdominal pain	1.98 (1.25–3.12)	0.004	–	–
Vomiting	1.39 (0.92–2.10)	0.139	–	–
Cough	0.76 (0.45–1.26)	0.284	–	–
Diarrhea	1.00 (0.60–1.66)	0.988	–	–
Petechiae	1.22 (0.70–2.10)	0.469	–	–
Rash	0.47 (0.17–1.34)	0.214	–	–
Epistaxis	1.08 (0.40–2.89)	0.800	–	–
Bleeding from the gums	1.00 (0.49–2.03)	1.000	–	–
Severe bleeding	25.76 (14.22–46.68)	<0.001	20.75 (10.66–40.38)	<0.001
Hepatomegaly	2.75 (0.68–11.15)	0.158	–	–
Pleural effusion	9.33 (3.53–24.66)	<0.001	10.23 (4.65–22.54)	<0.001
Rapid, weak pulse	∞ (not estimable)	<0.001	–	–
SBP < 90 mmHg	∞ (not estimable)	<0.001	–	–
DBP < 60 mmHg	18.01 (10.94–29.64)	<0.001	–	–
Pulse pressure < 20 mmHg	4.98 (1.77–14.04)	0.004	–	–
Hematocrit > 45%	1.41 (0.91–2.18)	0.133	–	–
WBC < 4000/µL	1.02 (0.67–1.56)	1.000	–	–
Platelet ≤ 100,000/µL	13.13 (6.27–27.48)	<0.001	3.62 (1.10–11.94)	0.035
Neutrophil > 70%	0.76 (0.48–1.20)	0.264	–	–
Lymphocyte < 20%	0.95 (0.61–1.48)	0.911	–	–
AST > 40 U/L	2.86 (1.76–4.63)	<0.001	–	–
ALT > 40 U/L	2.12 (1.40–3.23)	<0.001	–	–

Notes: OR = Odds Ratio; CI = Confidence Interval. Crude OR derived from univariable logistic regression. Adjusted OR derived from multivariable logistic regression using backward elimination. Variables with OR = ∞ indicates zero events in one group (complete separation); p-values are based on Fisher’s exact test for these variables. All p-values are reported to three decimal places, and p-values < 0.001 are shown as <0.001. Binary variables were generated based on clinically relevant cut-off values in accordance with WHO dengue guide lines and previous prognostic studies.

## Data Availability

Not applicable.
